# Fusion-Learning of Bayesian Network Models for Fault Diagnostics

**DOI:** 10.3390/s21227633

**Published:** 2021-11-17

**Authors:** Toyosi Ademujimi, Vittaldas Prabhu

**Affiliations:** Harold and Inge Marcus Department of Industrial and Manufacturing Engineering, Pennsylvania State University, University Park, PA 16802, USA; tta5@psu.edu

**Keywords:** fusion-learning, Bayesian Network, smart maintenance, fault diagnostics, natural language processing, technical language processing

## Abstract

Bayesian Network (BN) models are being successfully applied to improve fault diagnosis, which in turn can improve equipment uptime and customer service. Most of these BN models are essentially trained using quantitative data obtained from sensors. However, sensors may not be able to cover all faults and therefore such BN models would be incomplete. Furthermore, many systems have maintenance logs that can serve as qualitative data, potentially containing historic causation information in unstructured natural language replete with technical terms. The motivation of this paper is to leverage all of the data available to improve BN learning. Specifically, we propose a method for fusion-learning of BNs: for quantitative data obtained from sensors, metrology data and qualitative data from maintenance logs, corrective and preventive action reports, and then follow by fusing these two BNs. Furthermore, we propose a human-in-the-loop approach for expert knowledge elicitation of the BN structure aided by logged natural language data instead of relying exclusively on their anecdotal memory. The resulting fused BN model can be expected to provide improved diagnostics as it has a wider fault coverage than the individual BNs. We demonstrate the efficacy of our proposed method using real world data from uninterruptible power supply (UPS) fault diagnostics.

## 1. Introduction

The productivity and sustainability of manufacturing and service industries depend largely on prompt identification of fault root cause(s). Since these systems are complex and machine breakdowns are inevitable, fast and accurate root cause analysis (RCA) facilitates quick fault root cause identification as the bottleneck of machine repairs is fault diagnosis rather than the actual repair [1,2]. Bayesian Network (BN) is a widely used machine-learning algorithm for diagnostics in many fields such as manufacturing, healthcare, genomics, and social sciences. Key advantages of BN can be summarized as follows [3,4,5,6]:

- Interpretability—Clearly identifies relationship between variables;
- Root Cause Analysis—Can model the cause and effect relationship together with the causation path in case of indirect causation;
- Model Uncertainty—Since it is based on probability theory, it can readily handle uncertainty, which is inherent in fault diagnosis;
- Compact Representation—Directed acyclic graphs (DAG) are used to represent variables that influence each other along with causal direction.

In comparison to traditional fault diagnostics (FD) models such as fault tree analysis (FTA) and empirical models which are good at modeling less complex, well understood phenomena, the above advantages along with the less restrictive modeling assumptions of BN render it a better choice. For example, BN provides flexibility in the network structure [7] and number of variable states while FTA is limited to binary variables [8,9]. Additionally, a single BN model can be used for both diagnostic and prognostic [10] which is usually not the case with conventional FD models.

Learning in a BN model consists of two steps: (i) learning the structure of its DAG; and (ii) learning its parameters that model the degree of influence among its nodes, which are represented in the form of conditional probability tables (CPT). Finding the optimal DAG from observational data is NP-hard, thus heuristic search algorithms are employed [11,12,13]. The correctness of the resulting structure cannot be guaranteed as only the correlation between variables can be estimated [14,15]. Furthermore, because of technical and economic constraints, sensor data may not be able to cover all possible faults [1,16], therefore such BN will be incomplete [6]. Additionally, the causal sufficiency assumption which establishes the theoretical foundation to learn the DAG structure from observational data [15] can be violated if fault causes are incomplete. At present, the best way to learn the most suitable BN structure is by using expert knowledge elicitation, and its accuracy is limited by the expert’s memory, ability to deal with large networks, and inter-expert consistency [15,17]. To date, qualitative data has been rarely used to train BNs for fault-diagnostics even though many organizations store diagnostic knowledge in their software systems that can provide historic causation information; for example maintenance logs stored in CMMS (Computerized Maintenance Management System) software, corrective and preventive action report (CAPA) in Quality Management Systems (QMS) [18,19,20,21].

The motivation of this paper is to leverage all of the data available—quantitative data from sensors and qualitative data from maintenance logs—to improve BN learning in terms of the DAG accuracy and fault coverage. Since the two data types are disparate and cannot be directly combined to learn a single BN model, we propose a method in which we first generate individual BN model for each data type then combine the two BN models. We call this fusion-learning of Bayesian Networks. An appropriate feature engineering technique is applied to each data type before learning the BN from it. Our method utilizes the qualitative data to construct the BN DAG to leverage any causal information within it, potentially making expert knowledge elicitation easier.

The contribution of this paper is twofold: (1) we propose a human-in-the-loop natural language processing approach for expert knowledge elicitation of the BN structure aided by logged natural language data instead of relying on exclusively their anecdotal memory; (2) we propose a combined modeling algorithm for training BN from both qualitative and quantitative data sources that result in improved fault root cause identification when compared with BN trained using only one data source. The remainder of the paper is structured as follows. We provide some background into Bayesian Networks and review related research work. Next, we present the fusion-learning algorithm followed by a case study on uninterruptible power supply (UPS) system fault diagnostics to showcase its application. Finally, we offer our concluding remarks and discuss future work.

## 2. Background and Literature Review

A BN model represents the factorization of the joint probability distribution among a set of random variables. It can be denoted as a set (G,θ) where  G is the directed graph that depicts the dependencies between the variables and is made up of the vertex or node set V and the edge or arc set E. The parameter set θ represents the degree of influence between the nodes. For fault diagnostics applications, the nodes correspond to faults and a direct arc between two nodes such as $X_{j}\to X_{i}$ means that $X_{j}$ is the parent or direct cause of $X_{i}$, and $X_{i}$ the child or direct effect of $X_{j}$, denoting a probabilistic cause and effect relationship between the nodes. An indirect path between two nodes via another node such as $X_{j}\to X_{i}\to X_{h}$, means that $X_{h}$ is a descendant of $X_{j}$, and $X_{j}$ is an ancestor of $X_{h}$. The joint probability between a set of random variables X can be decomposed as follows using the chain rule of BNs:

(1) $$P\left(X\right)=\overset{r}{\underset{i=1}{\prod }}P(X_{i}|Pa\left(X_{i}\right))$$

where $Pa\left(X_{i}\right)$ is the parent set of variable $X_{i}$ and r is the total number of nodes. A BN model embeds a local Markov property that states that each variable is conditionally independent of its non-descendants given its parents. Although it is difficult to derive causality from observational data as statistical dependency does not always imply causality [22], the parent-child relationship is interpreted as causality for fault diagnostics. Training a BN model firstly entails learning the structure G followed by fitting the parameters θ from data set D to the learned DAG G, written mathematically as:

(2) P(G,θ|D)=P(G|D)×P(θ|G,D)

Recent trends in smart systems/Industry 4.0 have resulted in an increased use of aftermarket add-on industrial internet of things (IoT) sensors for equipment condition monitoring such as vibration sensors, temperature probes, cameras and so on. These sensor data are being used to train BN models for fault diagnostics. Score-based, constraint-based or hybrid methods are used to learn the BN structure from the data. Sensor data was used in [23] to identify any abnormal condition in electro-fused magnesia smelting process. For diagnosing the root cause of defective wafers, BN structure was generated from sensor data in [10] using K2 algorithm. Liu and Jin [24] diagnosed fixture fault in an automobile taillight assembly plant using part metrology data. Sensor data from machining process was used in [25] for diagnosing surface roughness faults, and in [26] to diagnose tool wear, workpiece hardness and stock size dimensional variation faults. The reader is referred to [4,5] for a review of BN and machine learning algorithms used for fault diagnostics.

Methods to learn the DAG from observational data is based on the faithfulness and causal sufficiency assumptions. Faithfulness assumption ensures equivalence between the independence relationships in both graph structure and the underlying joint distribution. Causal sufficiency assumption assumes that all variables are present in the data. Both of these assumptions could be violated by using only one data source as not all variables can be measured using sensor data only. Moreover, at best, only correlation between variables can be discovered, which is the essential graph (skeleton and v-structures), as observational data cannot distinguish between two graphs having the same skeleton (undirected graph) and v-structure [14]. This can result in the discovery of a set of “equivalent class” networks rather than the full causal relationships between variables [3].

More often than not, the findings of fault investigations are documented by humans in the form of a report or note written in natural language. Since several personnel are responsible for this document writeup at different times, misspellings, abbreviations and colloquial terms of choice are used as there is usually no standard terminology or controlled verbiage to follow. This unstructured form of the data makes it difficult to reuse and process automatically using software [20,27], but reusing them can potentially save diagnostics time. To this end, Brundage et al. [27] proposed a framework for automated storage and retrieval of diagnostics knowledge to facilitate their reuse in future fault diagnostics. 

CAPA reports and maintenance logs are two widely used documents for recording problem solving steps/outcomes in industry. These documents contain several data entry fields. The two most important fields apart from the equipment identification field are: (1) problem description field that states the issue that was investigated and (2) the resolution field that describes how the problem was resolved. The field names vary but the information they contain is usually the same. See example of maintenance log and CAPA report entry fields in Table 1 and Table 2. The “Problem Description” field entry may contain data written in natural language by human operators such as “high temp alarm”, “high temperature alarm”, “temperature is outside of its limit”, or semi-structured automated sensor message such as “high temperature threshold violation alarm”, while the “Resolution” section contains human generated raw text such as “air conditioning unit is switched off”, “ac unit is off”, “a/c was switched off” and so on. These verbiage inconsistencies in the data make it difficult to directly generate a BN from the raw data as it can result in incorrect CPT values. Although few companies do provide the option to utilize prepopulated drop-down menus for maintenance log data entry as a way to control the vocabulary, not all problem and resolution descriptions are usually included in the menu, and as such, still require a comment section where such descriptions are documented.

To improve the quality of maintenance data in order to make it fit for reliability analysis, Hodkiewicz and Ho [21] proposed a rule-based method with rule sets constructed by domain expert as a list of “if condition perform action” statements, meaning that new rules will need to be created for each situation encountered and the rules could grow fast in complexity. Natural language processing (NLP) technique is another approach being applied. To structure maintenance log data in order to predict the criticality and duration of a maintenance issue using neural network, Usuga Cadavid et al. [28] applied a feature-based NLP approach called CamemBERT. Sexton and Fuge [29] applied NLP to recover a structured representation of a system’s engineering knowledge from unstructured maintenance work order data. A methodology that uses text analytics techniques in combination with human-assisted thesaurus development to convert maintenance log data into a knowledge graph was proposed in [30]. Rajpathak et al. [31] utilized an integrated framework for automatic creation of a domain ontology for fault detection using warranty repair data of an automotive OEM. The technical tags in the data were classified as either symptom, part or action. Human in the loop *n*-gram data tagging method was utilized in [19,20,32] where an expert first tags the keywords before classifying the tags as either problem, item, or solution. This hybrid approach of labor intensive expert manual tagging and subsequent automated tagging is best suited to maintenance log data [20]. In contrast to traditional NLP applications such as named entity recognition or POS tagging that have very large corpora of documents for which several NLP libraries (such as SpaCy, NLTK, and so on) have been built to automate the process, maintenance data are usually much fewer (less than 10,000 rows) [33], as equipment failure is a relatively rare event. Additionally, the logs contain domain specific technical terms that traditional NLP pipelines fall short of processing [33]. For instance, the NLP stop words removal step, where common words such as “not” or “no” are removed, can completely reverse the contextual meaning of a phrase; for example, instead of having “power is not out” or “There is no power outage” which are correct, we get “power is out” or “There is power outage”, which are both incorrect. 

The underlying assumptions in the NLP data tagging methods above is that each row of data only maps to one problem and solution (or symptom and action) and thus fails to capture the chain of causation of a fault where more than a single direct cause is present. For example, “device down” problem or symptom could be caused by “power failure” which in turn could result from “transformer fire”. This series of events is valuable information in the root cause analysis process and we create a method to capture this information using BN in this paper.

There are many applications in the healthcare domain that utilize text data to generate BN for medical diagnostics such as the work of Raghuram et al. [34]. The use of controlled vocabulary has the potential of improving the quality of qualitative data. Taxonomy, thesaurus and ontology are types of controlled vocabulary also referred to as semantic models. They contain domain specific terms including their synonyms or preferred label in the form of concepts, with taxonomy being the simplest variant and ontology the most sophisticated. BN has been generated from ontology in several domains such as threat prevention [35], esophagus cancer diagnostics [36] and emergency event reasoning [37]. 

In manufacturing domain, Sayed and Lohse [38] proposed using an extended version of the product-process-equipment design ontologies integrated with failure mode and effect analysis (FMEA) information to construct the DAG. Extracting cause-effect relationship from equipment’s maintenance manual was proposed in [39]. De et al. [40] generated an ontology relationship diagram from both FMEA and corrective action report data before converting it to a BN used for reducing detection-to-correction time. Kurscheidt et al. [7] proposed a process mining methodology to discover BN from structured simulated maintenance event logs and proposed using real unstructured text data as future work. Only a few researchers have created BN from ontology in manufacturing domain. This is partly because creating an ontology itself is not an easy task and most approaches proposed in the literature have had limited practical success due to lack of scalability and interoperability, resulting in limited domain-wide acceptability [41,42].

To improve diagnostics performance, multi-source information fusion has been proposed by several authors, such as BN based multi-sensor fusion for improved fault diagnosis of vectoring nozzle system [43]. For improving the condition-based diagnostics of vehicles, Bayoumi et al. [44] proposed a methodology to integrate maintenance data and sensor data by tagging events from both data sets with location, severity, and rarity parameters. For creating a BN fault diagnostics model, Nguyen et al. [1] exploited historical data of unobserved equipment components to reduce the search space of potential faulty components. The proposed methodology is, however, limited to diagnosing product quality faults observed when measuring parts and the historic data used only contain information about the binary state of the machine with no information about the specific machine fault. Recently, natural language log data was integrated with sensor data in a CNC machine tool degradation experiment and the two data types exhibited high correlation [45].

There are several reasons for combining multiple BNs into a single BN. One reason might be to improve modeling performance of the BN by fusing alternative models together. Another reason might be for knowledge integration of either a large domain BN consisting of several subset domains each with its own subject matter expert, or disparate data types that cannot be merged into a single dataset that require the individual BNs to be learned separately. The latter is the case in this paper. To improve the BN structure learned from numeric data, Kim and Cho [46] developed a method that generates multiple BN structures using genetic algorithm followed by selective combination of the resulting BNs for adaptive prediction. A greedy constructive search algorithm for finding a topological ordering that is suitable for guiding the fusion process was proposed [47]. Their method was applied to combine BN models of supermarket sales from three different locations where the dataset used to construct each BN is not available to the others. Pẽna [48] presented a heuristic method of creating a consensus DAG from several DAGs provided by multiple experts that only represents independencies agreed upon by all the given DAGs and has the fewest parameters possible. Their algorithm is, however, only applicable to aggregating BNs defined over the same set of variables.

A method of combining different BN that preserve both the conditional independencies of each BN and the characteristics of the individual BN parameters was proposed in [49]. Although their method is applicable to BN with different variables, they make the assumption that an ancestral ordering that helps to prevent acyclicity exists which is not always the case. Tabar and Elahi [50] extended the method in [49] to include cases where an ancestral ordering is not present by using simulated annealing algorithm to obtain an acyclic graph in which the minimum arcs have been removed. To develop a BN for a large domain, Del Sagrado and Moral [51] studied the consensus model that would be obtained by aggregating the knowledge provided by several experts who are specialists in some subset of the whole knowledge domain via the union and intersection of the independencies depicted in each sub graph. All the above methods are limited to BN learned using quantitative data only. To improve the performance of BN for chiller fault detection and diagnosis, Wang et al. [6] proposed fusing sensor data with multi-source non-sensor data. Their approach is limited in that the maintenance record data used only contains binary information on whether or not maintenance was carried out and they utilized a fixed restrictive three-layer DAG structure which is not generalizable.

## 3. Fusion-Learning of Bayesian Network Models

**Figure 1:**
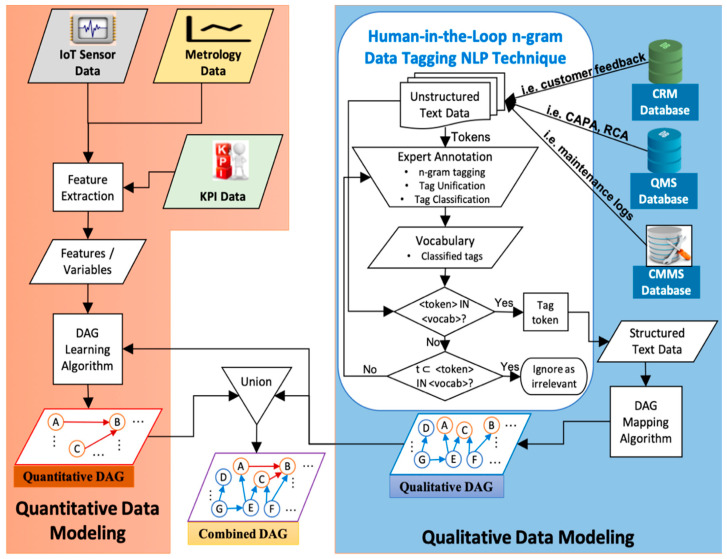
Fusion-learning flowchart.

We propose a generic approach to leverage a multitude of heterogenous data sources by first developing a BN model for each data source individually and then fusing these BN models together. Furthermore, we propose a data tagging algorithm to generate a BN from the quantitative data sources. The schematic of the fused learning framework is presented in Figure 1. It entails generating the qualitative DAG first from the text data sources using human-in-the-loop data tagging NLP technique followed by incorporating the qualitative DAG’s edge information as constraints into the BN structure learning algorithm to generate the quantitative DAG. Lastly, the combined DAG is obtained by taking the union of the two BN structures generated from the two disparate data types, and the parameters are directly learned from both types of datasets. Unlike the quantitative data, the conventional structure learning algorithms are not applicable to the qualitative data as the data already contains the causal relationship information which need to be mined instead. Fusing the qualitative DAG into the quantitative DAG learning process ensures acyclicity in the final combined DAG and eliminates relearning of any quantitative edge already learned in the qualitative data modeling. For this algorithm to work, however, the common variables in each model must have the same names.

### 3.1. Qualitative Data Modeling

Human-in-the-loop data tagging technique, which is a hybrid of fully manual and automated tagging, is proposed for the generation of the qualitative BN DAG. Manual tagging of the whole data row by row is very labor intensive as it does not take full advantage of the similarities between related events with same keywords but slightly different wordings. On the other end, fully automated tagging using some classification machine learning algorithm is most applicable to domains with larger datasets. Thus, a hybrid method where a subset of the data is tagged first to generate a vocabulary of terms followed by the extraction of other rows that have same keywords from the corpus is best. This data structuring process entails cleaning and condensing the long natural language text into unique meaningful standardized format using *n*-gram data tagging. After the tags have been generated, they are classified into their BN hierarchical level variable categories. The whole process can be divided into the expert annotation step, tag extraction step and finally BN mapping step.

#### 3.1.1. Expert Annotation

**Figure 2:**
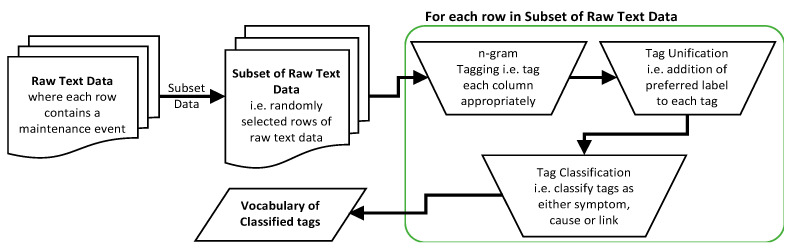
Expert annotation step flowchart.

The flowchart for the expert annotation is presented in Figure 2. This step involves creating a vocabulary from a subset of the text data via manual keywork tagging followed by the unification of all instances of each unique fault and then tag classification. Keyword tagging is a method used to annotate a corpus with meaningful terms chosen freely by the expert [52]. Each tag is a list of n sequential words, known as *n*-gram and n≥1, that represent the important keywords in the document. The appropriate *n*-gram tag is applied to each maintenance event type such that its contextual meaning is preserved and uniquely defined, thus unambiguous. To unify all instances of each unique fault in the whole dataset that have different tags either because they were written using different wordings, abbreviations or misspellings, tag unification is implemented by assigning a preferred label to the tags in the vocabulary. Each tag is further classified as either symptom, cause or link. Symptom category represents the fault that was observed that triggered the maintenance event while the cause category represents the identified underlying reason the fault occurred. Link category preserves the causal direction in the case where several causes were identified that form the chain of causation. Each row of maintenance data will typically have one symptom tag but could have more than one cause tag depending on how many causes are listed. Rows that have only one cause tag will have no link tag as those with m cause tags will have m−1 link tags. The cause tags in a row are arranged in an order according to how they occur in the document and the link tag preserves the direction of causation.

#### 3.1.2. Tag Extraction

**Figure 3:**
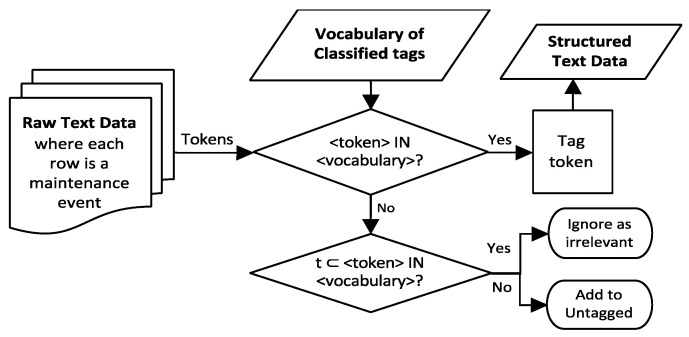
Tag extraction step flowchart.

The vocabulary created in the annotation step is used to extract the keywords from the raw maintenance data. Since the vocabulary contains only a subset of tags present in the data, the untagged tokens are recycled back to the expert for further annotation. This manual tagging by human followed by automated tagging sequence iteration is usually not a one-time cycle and should continue until all the useful data in the corpus have been completely tagged. Since manual tagging requires lots of manhours to complete, this hybrid approach is more efficient. Using a tag ranking rule such as TF-IDF (term frequency and inverse document frequency) metric to present the most important words to the expert tagger as used in the work of Sexton et al. [20] could speed up the manual tagging process in some cases. The workflow for the tag extraction step is shown in Figure 3.

#### 3.1.3. Bayesian Network Mapping

**Figure 4:**
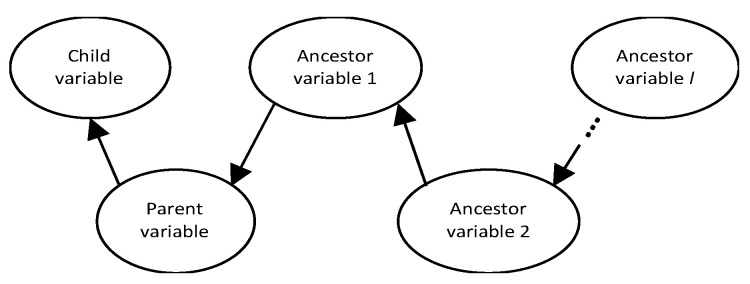
Generic BN structure for the tagging output.

**Figure 5:**
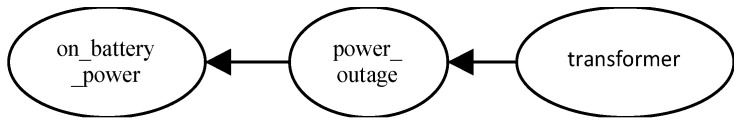
Corresponding Bayesian Network graph for example in Table 3.

In this step, the classified tags are mapped to their corresponding BN categories of child variable, child state, parent variable, parent state, ancestor variable 1, ancestor state 1, …, ancestor variable l, ancestor state l; where l is the number of ancestor levels present in a particular log entry. The corresponding generic BN structure is shown in Figure 4. The number of hierarchy levels in each row of data varies and some data only map to the child and parent classes. Symptom tags map directly to the child variable while the cause tags map to parent and ancestor variables. When multiple cause tags are present, the link tag is used to determine which is parent versus ancestor level. Table 3 shows an example of our *n*-gram tagging process output including classes of each tag and the corresponding BN class. Note that the problem field header here says “Short Description” which usually maps to the child variable/state, while the resolution field maps to the cause(s). As can be seen in this example, an *n*-gram tag might need to be split into variable and variable state such as in the case of “transformer_fire” being split into “transformer” which is the variable name and “fire” which is the variable state. The state of other variables might not be explicitly included in the tag but inferred such as binary variables i.e., yes or no states, such as the parent state in this example. The corresponding BN structure for the data in Table 3 is presented in Figure 5. If a link tag is not included and there are two cause tags present, then the second cause tag is the parent of the first cause tag. Because maintenance investigations are usually triggered by measured numeric data going out of bounds, quantitative variables are usually the children of qualitative variables. Finally, the learned DAG is named the qualitative or text DAG $G_{text}$ which contains the vertex and edge sets $\left(V_{text},E_{text}\right)$. This qualitative DAG is then utilized in learning the quantitative DAG in the next section.

### 3.2. Quantitative Data Modeling

Once $G_{text}$ has been created, the quantitative DAG can be constructed in a two-step process of feature extraction followed by DAG learning. 

#### 3.2.1. Feature Extraction

In this step, the raw time series sensor data is converted to time domain and/or frequency domain features using signal processing techniques. For sensors preinstalled by the original equipment manufacturer (OEM), the quantitative data might already be in the form of messages or alerts, and further feature extraction might not be necessary in this case.

#### 3.2.2. DAG Structure Learning

The quantitative or numeric DAG $G_{num}$ can be learned using any BN structure learning algorithm such as expert elicitation or data driven heuristic search methods. The arc set information already learned in the qualitative DAG is incorporated into the quantitative DAG learning process in two ways: (1) they are added as constraints to prevent cyclicity in the merged final combined DAG and (2) used to determine the edges between pairs of quantitative variables that have already been determined in the qualitative DAG. The former can be implemented either as additional criteria for selecting the quantitative structure by choosing only candidate networks that do not result in cyclicity in the final combined DAG given the qualitative DAG, or as a rule to resolve cyclicity if it occurs by reversing the quantitative edge direction instead of that of the qualitative edge. The later can be implemented in most data driven structure learning algorithms by adding the edges as “whitelists”. Both of these can help improve the learning process by reducing the DAG search space. Since the maintenance log data contains historic ground truth causal relationship, incorporating the qualitative DAG information into the quantitative DAG learning process improves the resulting BN DAG.

### 3.3. Model Fusion

The fused model structure is obtained by taking the union of the two individual BN DAGs. i.e., $G_{comb}=G_{text}\cup^{\;}G_{num}$, and the pseudocode is presented in Algorithm 1. Since the two DAGs were not created independently from one another, there will not be any conflicting edge directions that need to be further resolved at this stage. Since both data types are available, the combined parameters can be derived directly by first unifying them using their date and time stamps before estimating the CPTs. This approach eliminates the error associated with approximating the combined BN CPT from the individual networks’ CPTs.
**Algorithm 1.** BN DAG model fusionData:
$G_{text}=\left(V_{text},\;\;E_{text}\right);\;G_{num}=\left(V_{num},\;\;E_{num}\right)$
Initialize:
$V_{comb}=V_{text},\;\;E_{comb}=E_{text}$
for vertex in $V_{num}$
 if $vertex\notin V_{comb}$
  Add vertex to $V_{comb}$
for edge in $E_{num}$
 if $edge\notin E_{comb}$
  Add edge to $E_{comb}$
return $G_{comb}=\left(V_{comb},\;\;E_{comb}\right)$



## 4. Case Study

To demonstrate the application of our method, we apply it to train a BN using real-world data. The case study involves developing a BN model for the information technology (IT) department of an organization for diagnosing uninterruptible power supply (UPS) units’ faults. The organization owns several UPS units used as backup power for network switches. These UPSs are integral to keeping the network running and to avoid denial of service whenever there is temporary power outage. There are about 300 units in different physical and geographic locations within a state in the U.S., making it unrealistic to monitor each one physically. As such, online remote monitoring is employed instead. These devices also act as sort of a surge protector by monitoring the characteristics of the input power using preinstalled voltage and current sensors so that it can provide adequate compensation when needed to prevent electrical damage to the switches. The UPSs are also equipped with several other internal sensors for monitoring the health of the battery and an external sensor to monitor the environmental temperature. Each UPS is fitted with a network management module/card that interfaces it to an online monitoring system where the state of all the sensors are constantly monitored. 

Whenever an alarm occurs, a work order is opened to determine the root cause in order to rectify the problem. The troubleshooting process, as well as the final resolution are documented in an incident log written in natural language by the experts. Since quick resolution is highly desired to get the network up and running, we apply our method to create a BN for diagnosing the faults by utilizing the incident log data that can potentially speed up fault diagnostics. A brief description of the data, including the column header titles, data type contained in each column including its data content is presented in Table 4. The “Short Description” section contains the UPS asset number along with the error message being observed, documented by a human operator. This error message is from a sensor and thus is numeric data in a semi-structured format as different UPSs have their unique message predefined by the OEM. The “Comment and Work Notes” column contain the details of the troubleshooting actions taken including the date it was performed and the outcome of the actions. This section is usually very lengthy as it sometimes takes a couple of tries before an incident is resolved. The final step taken to resolve an incident is usually documented in the “Resolution Notes” column but the root cause documented in the “Resolution Notes” is sometimes incomplete.

**Figure 6:**
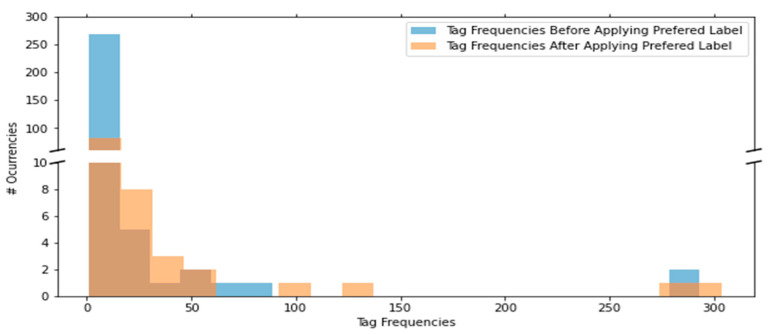
Frequency distributions of raw data tags before tag unification vs. unified tags after applying preferred labels.

We begin creating the BN by first generating the qualitative BN model from the incident logs. The logs cover a duration of about a total of three years and the current maintenance policy is run to failure (reactive maintenance) where a UPS is repaired after it fails. Each row is a unique incident and the columns of interest are the “Short description” and “Comments and work notes” columns where discussions on the observed alarm and possible problem causes are documented. The data was tagged using our proposed *n*-gram data tagging NLP technique. The extracted tags were classified as either symptom, cause or link before being grouped into the appropriate BN taxonomy of parent variable/state, child variable/state, and ancestor variable/state. A preferred label was also assigned to each tag to unify tags with same meaning but different wordings including misspellings. The reduction in the number of low-frequency tags and corresponding increase in that of high frequency tags resulting from the tag unification is displayed in Figure 6. Two density plots of before and after applying the preferred labels are plotted on top of one another. The number of low frequency tags such as those that occur only once or twice in the corpus reduced as they were replaced with a preferred label. A few examples of low frequency tags and the preferred label tags they were replaced with is presented in Table 5. “management_module” tag which is a low frequency tag that only occurred once in the corpus was replaced with its preferred label “Management Card”. As such, the number of occurrences of low frequency tags such as “management_module”, “ups_card” and “mgmt._card” reduced while the number of occurrences of high frequency tags such as “Management Card” increased from 19 to 22 occurrences. A significant advantage of this unification step is that it prevents the underestimation of probabilities of individual events with different raw data tags thus making the estimated CPT tables more representative. To illustrate this, if we take a subset of the data which contains only two events presented in Table 5 and calculate the marginal probabilities of each event. Assuming each event has two states, “yes” vs. “no” for “high_temperature_alarm” and “good” vs. “faulty” for “management_card” such that we have the states “yes” and “faulty” respectively whenever the corresponding tag appears. Using the raw data, the probability of observing a “high_temperature_alarm” is 4/62=0.065. Upon unification, the probability increases to 40/62=0.645. 

Tagging the whole 733 rows of raw data using our method took about 20 min per row. This long duration was due to the very lengthy “Resolution Notes” and “Comment and Work Notes” columns of the data. Additionally, only 421 rows were required to be fully manually tagged to complete the whole 733 rows as the rest were extracted which took 3 iterations between fully manual tagging and automated tag extraction. The reader should note that this number can vary significantly as it depends on the initial subset of raw data randomly selected as well as the number of words present in each row. Examples of some incidences and their corresponding tags are presented in Table 3 and Table 6. Incidences that were not useful for diagnostics purposes such as “no fault found” and “unknown resolution” incidences, as well as routine maintenance requests were discarded in this analysis. The total number (rows) of remaining tagged incidences was 429 and the highest occurring tags are presented in Table 7. All of the observed faults which are the child variables of the BN are alarms from sensors in this case study because an error message is what triggers the investigation process. This could be different in other industries where technicians can initiate work orders based on manual observation of machines not performing as expected.

**Figure 7:**
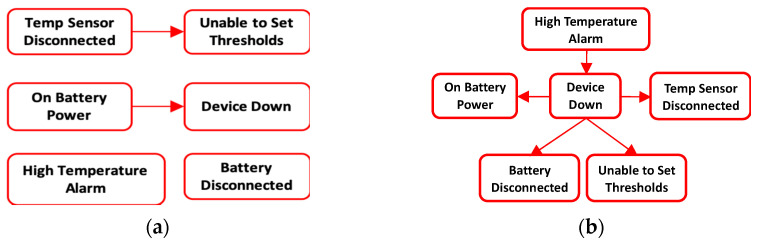
BN DAG structure for the top six occurring UPS messages. (**a**) Correct quantitative BN model DAG obtained from Expert opinion and qualitative data; (**b**) Incorrect quantitative BN model DAG obtained from Hill Climbing score-based BN algorithm.

Next, the quantitative BN was constructed using the sensor data and acyclicity constraints from the qualitative DAG. Since the sensors in this case are factory fitted, their output is already formatted by the original equipment manufacturer into specific error messages. Thus, no further feature extraction was required. The error messages were retrieved from the “system log” file. Expert opinion was used to generate the quantitative BN structure and added arcs did not result in violation of the final combined arc acyclicity. The quantitative DAG of the six highest occurring faults is displayed in Figure 7a and it only contains two edges. The edge between “Temp Sensor Disconnected” and “Unable to Set Thresholds” was obtained from the qualitative data DAG. For comparison, both score-based and constraint-based heuristic search methods were applied to learn the quantitative DAG for the top 6 occurring faults and the obtained DAG using the Hill climbing (HC) score-based method is presented in Figure 7b. The search algorithms were implemented using bnlearn package [53] in R software. The HC DAG, similarly to the DAG obtained from the other search algorithms, had a structural hamming score (SHD) value of 5. The SHD score counts the number of incorrect arcs and a score of zero means that the learned DAG is the same as the correct one. The poor performance of the heuristic search BN learning methods agrees with the BN application literature that expert opinion is usually the most accurate method to learn the DAG for real life applications. Estimating the DAG using heuristic search methods required large amounts of balanced class data which is uncommon in real life applications. Since failure events are much rarer than healthy state, there is class imbalance between them. The qualitative data which contains expert opinion aids in the DAG generation particularly in situations where experts with years of experience that can easily elicit the DAG structure have retired or left the organization. The DAG can be easily generated from the qualitative data using our proposed method.

**Figure 8:**
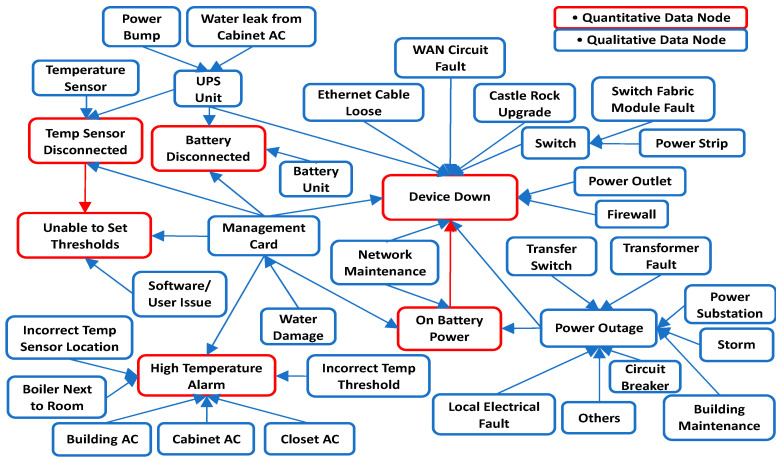
Fused Bayesian Network structure for top six occurring UPS messages.

**Figure 9:**
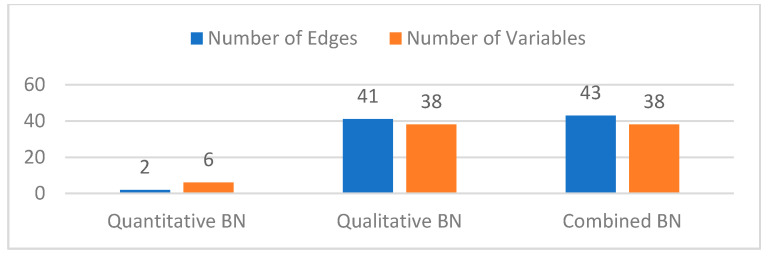
Comparison of the number of variables and arcs of the BN model in Figure 8.

Finally, the two DAG structures were combined by taking their union, and the final DAG for the top six occurring UPS messages is shown in Figure 8. Note that in this figure, the qualitative DAG is the whole DAG without the red colored quantitative edges. The combined model parameters were then estimated from both data. It can be seen from the results that analyzing a single data type leaves out relevant edges and variables in the model (see Figure 9). The quantitative DAG has 2 edges and 6 variables while the qualitative DAG has 41 edges and 38 variables. Since both qualitative and quantitative variables are present in the qualitative data, the combined DAG has 43 edges and 38 variables in total. Thus, the maintenance log data contains a richer set of fault variables than the sensor database. Although only two edges are missing in the qualitative BN model, this number can get larger for models with more quantitative variables. From the quantitative model (Figure 7a), the root cause for “Device Down” fault is “On Battery Power” which is not the actual root cause. From the combined model, however, we can see that the root cause of “Device Down” could be either a Faulty Circuit Breaker (i.e., BN variable “Circuit Breaker” with state “Faulty”) or issues with the “Management Card” and so on. Therefore, we get improved root cause analysis by fused modeling. 

**Figure 10:**
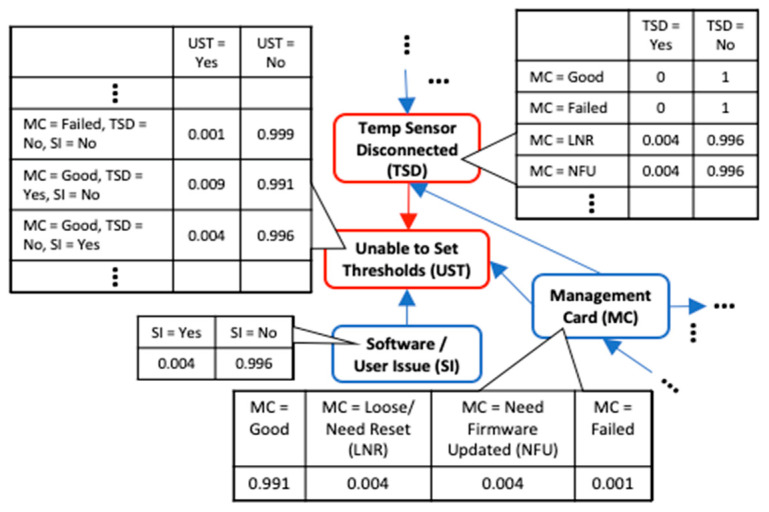
Detailed BN for “Unable to Set Thresholds” fault showing its direct parents only and CPT tables.

Likewise, in using the qualitative data model to diagnose “Unable to Set Thresholds” fault, we miss the fact that a disconnected temperature sensor (“Temp Sensor Disconnected” message) can cause this error further making the case for the need to include both data types to get a more complete BN model as no single data source sufficiently covers all possible fault modes. Consider a case where the “Unable to set Thresholds” fault exists in one of the organization’s offsite locations. The detailed BN for this fault is presented in Figure 10. Since the IT department is staffed in the main office, resolving this will require a technician to travel to the site with the right replacement equipment which they usually order from the OEM. If the quantitative DAG is used to diagnose this fault, then the only root cause is that the Temperature sensor is disconnected which occurs 63.6% of the time. Based on this, the technician might order a new temperature sensor probe if we assume he knows that a “temperature disconnected” message might mean that the temperature probe needs replacement. However, from the combined DAG, the root cause can also be faulty Management card 9.1% of the time or software issues 27.3% of the time. This means that there will be delayed downtime due to misdiagnosis 36.4% of the time which could be a lot of lost time considering that a new management card might have to be ordered first. The time to wait for the new part including the technician’s travel time for the second time is all lost time due to a misdiagnosis using the quantitative BN. This could delay repairs significantly possibly by days or weeks which will increase equipment downtime. Additionally, fused modeling allows for inferring additional information about sensor states that would have being impossible otherwise. For example, a “Temp Sensor Disconnected” message could be caused by a failed temperature sensor (“Temperature Sensor” node with state “Failed”) or failed UPS (“UPS Unit” node with state “Failed”) or the sensor being disconnected (“Temperature Sensor” node with state “Unplugged”). The only possible cause for this message if we modelled the quantitative data by itself would be that the sensor is disconnected thus leaving out these other plausible causes. Moreover, to illustrate how the qualitative data can corroborate the quantitative DAG edge information, although the edge between “Temp Sensor Disconnected” and “Unable to Set Thresholds” can be inferred from the sensor data based on a correlation between the two variables, the incident log corroborates the presence of this edge.

**Figure 11:**
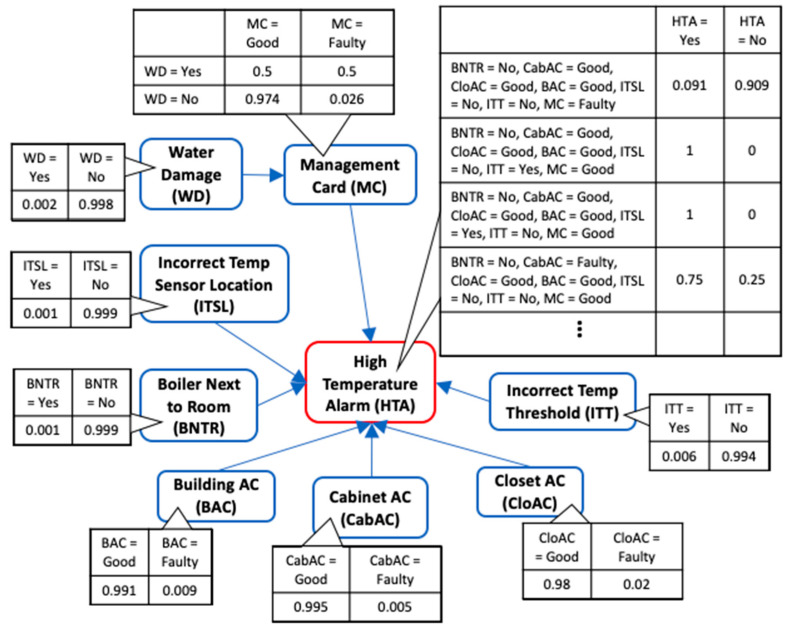
BN for High Temperature Alarm with binary variables using Fusion-learning.

**Figure 12:**
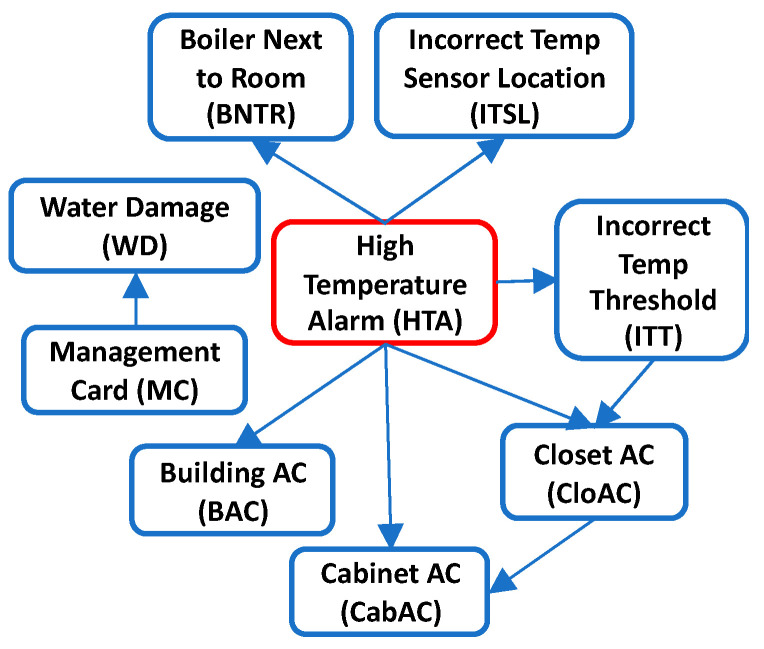
DAG structure for High Temperature Alarm learned using HC algorithm with binary variables.

Since it is impractical to show the complete BN along with its CPT due to its very large size, we present the BN DAG structure and CPT for High Temperature Alarm (HTA) fault with binary states in Figure 11. Only a partial CPT for variable “HTA” is displayed. Using the BN model, we can compute the probability of an event given some evidence. For example, the probability of observing HTA fault given that the Closet ac is faulty is 0.934. Likewise, the probability of observing that the Closet ac is faulty given that HTA fault exists is 0.404. The DAG structure for diagnosing HTA fault learned using HC algorithm is also presented in Figure 12 and has a SHD score of 9. Taking a closer look, it can be seen that the arc directions are mostly reversed which is not surprising given that correlation between variables can be learned at best using heuristic search methods. The misdiagnosis implication of the reversed causal direction is detrimental to the objective of improving the fault diagnostics using BN. This further validates our method as a much better alternative to generate the correct BN DAG for fault diagnostics.

## 5. Discussion

Due to the nature of the maintenance process, root cause analysis is usually manually conducted by experts and the sensor data mostly indicate that something is wrong but cannot always pinpoint the exact component or subcomponent responsible. There are cases, however, where the sensor data might be able to pinpoint the fault root cause such as frequency analysis of vibration sensor showing whether it is the bearing inner race or outer race that is damaged. Even though this might be a root cause, there might be other reasons why the bearing got damaged in the first place such as lubrication contamination or someone forgot to add lubrication. As such, building a BN model using the numeric data only leaves out valuable diagnostics knowledge and only fused learning of all available data paints a more complete picture of the reason for a fault occurrence. Furthermore, from a statistical perspective, observed sensor data being an exploratory data source can only be directly interpreted as correlation. Since we are interested in fault diagnostics and the qualitative data provides ground truth historic fault resolution that worked in the past, it provides causation information between variables. Using the maintenance log to generate the BN is a great aid for the expert who might not be able to remember all the causal relationships/variables when asked to elicit the DAG from memory, especially for BNs with a large number of variables and edges. Therefore, using the maintenance log, which is a more data driven approach, augments the expert BN generation process. The human-in-the-loop data tagging method proposed provides much flexibility in choosing the variable names and their states, as well as the number of layers of the BN.

In the present case study, all of the sensor faults are also documented in the incident log data along with their root causes as sensor messages are usually the triggering events for fault incident investigations. In some other cases where the sensor can report the exact root cause of the fault, if this root cause is the same as the one reported in the qualitative data, that is complementary information. However, in the case where there is a conflict in the reported root cause between the sensor data and incident log data, the sensor root cause will be treated as a false positive since the qualitative data is domain expert documented “ground truth” resolution carried out to eliminate the anomaly.

One major assumption here is that the qualitative data is of good quality such that it contains correct historic diagnostics knowledge. This might not be true for all organizations especially in those where technicians purposely document incorrect diagnosis for fear of being “replaced”. Other organizations may also have maintenance log data with sparse incorrect entries with little to no diagnosis data. It is also possible for misdiagnosis to occur sometimes. However, many companies have a quality system in place where change notices are issued for correcting such errors when they are discovered. Additionally, only fault root causes documented in the maintenance log data can be modeled.

## 6. Conclusions

The most prevalent approach of using numeric data only (i.e., sensors) to train BN models for fault diagnostics is deficient in that it leaves out many fault modes that are only available in qualitative data forms i.e., maintenance log data. The unstructured nature of the qualitative data is the main impediment to its usage. Therefore, in this paper, we proposed the fused modeling of all of the available numeric and text data to train Bayesian Network for improved fault coverage. Owing to the two data types not readily fusible due to their disparate nature, our proposed approach separately generates the individual BN before combining them. The text data is structured and converted to a BN using data tagging NLP method, and the obtained DAG is incorporated into any DAG learning algorithm to determine the quantitative DAG before the two DAGs are consolidated by taking their union. The method was then applied to generate a BN model for fault diagnostics of uninterruptible power supply (UPS) units.

Future work will involve the further application of the proposed method on a broader variety of manufacturing and service industries’ maintenance data. Furthermore, creating an automatic text structuring algorithm that automatically generates the fault diagnostics BN from text data will also be valuable future research.

## Figures and Tables

**Table 1:** Example of typical maintenance log data entry fields.

Input Field	Data Type	Structure
Incident or Problem ID	Integer	Structured
Asset ID	Integer	Structured
Equipment Name	Controlled Text	Structured
Date Opened	Time and Date	Structured
Problem Description	Raw Text or Automated Sensor Message	Unstructured/Semi-structured
Resolution Status	Binary Text or Automated Sensor Message	Structured
Resolution Notes	Raw Text	Unstructured
Resolution Date	Date and Time	Structured

**Table 2:** Example of typical CAPA report data entry fields.

Input Field	Data Type	Structure
Title	Raw Text	Semi-Structured
Equipment/Product(s) Affected	Integer/Controlled Text	Structured
Corrective Action	Controlled Text	Unstructured
Preventive Action	Raw Text	Unstructured
Date	Time and Date	Structured
Status	Binary Text	Structured
Resolution Notes	Raw Text	Unstructured
Resolution Date	Date and Time	Structured

**Table 3:** Example of the raw data, classified tags and BN mapping for one row of data.

**Raw Data**	**Short Description**		**Resolution Notes**			
	On battery power		Power outage due to transformer fire			
**Classified Tags**	**Symptom**		**Cause(s)**		**Link**	
	on_battery_power		power_outage, transformer_fire		due_to	
**BN Mapping**	**Child Variable**	**Child State**	**Parent Variable**	**Parent State**	**Ancestor Variable**	**Ancestor State**
	on_battery_power	yes	power_outage	yes	transformer	Fire

**Table 4:** Column titles of the raw data along with description of the data it contains.

Section Title	Data Type	Description	Form	Example
Number	Text + Number	A unique identifier for the incident	Structured	“INC0000049”
Short Description	Text + Number	UPS identifier and error message	Semi-Structured	“XX.Location-UPS01 high temperature threshold violation exists …”
Created	Date	Incident creation date	Structured	6 June 2017 11:23:01 a.m.
Resolution Notes	Text	Final resolution that resolved the issue	Unstructured	“UPS management module replaced. Correct temperature now reported by UPS”
Comments and Work Notes	Text	Notes containing the full troubleshooting process at each step including dates the troubleshooting step was performed	Unstructured	“21 June 2017 10:55:12 a.m.—Tech1 UPS management module replaced. Correct temperature now reported by UPS. 20 June 2017 10:49:18 a.m. Tech 2 Tech1 was onsite last week with a replacement temp sensor. Through testing, we determined the universal I/O port #1 is faulty… “

**Table 5:** Example of increase in density of low frequency tags resulting from addition of preferred label.

Raw Data before Applying Preferred Label		Corresponding Unified Tag after Applying Preferred Label	
Tags	Number of Occurrences	Tags	Number of Occurrences
high_temp_threshold_violation	2	High Temperature Alarm	40
high_temperature_threshold_violation	21		
high_temp_alarm	10		
high_temperature_alarm	4		
over_temp	1		
high_temp	1		
high_temp_violation	1		
management_module	1	Management Card	22
management_card	19		
ups_card	1		
mgmt._card	1		

**Table 6:** Two examples of raw data rows and their corresponding structured output.

**Raw Data**	**Short Description**			**Resolution Notes**				
	On battery backup power			… power is out, breaker tripped and …				
**Classified Tags**	**Symptom**			**Cause**			**Link**	
	on_battery_backup_power			power_is_out, breaker_tripped			none	
**BN Mapping**	**Child Variable**		**Child State**	**Parent Variable**		**Parent State**	**Ancestor Variable**	**Ancestor State**
	**Label**: on_battery_backup_power	**Preferred Label**: on_battery_power	yes	**Label**: power_is_out	**Preferred Label**: power_outage	yes	breaker	tripped
**Raw Data**	**Short Description**			**Resolution Notes**				
	Device down			UPS was replaced				
**Classified Tags**	**Symptom**			**Cause**			**Link**	
	device_down			ups_was_replaced			none	
**BN Mapping**	**Child Variable**		**Child State**	**Parent Variable**		**Parent State**		**Ancestor Variable/State**
	**Label**: device_down	**Preferred Label**: device_down	yes	**Label**: ups	**Preferred Label**: ups_unit	**Label**: was_replaced	**Preferred Label**: failed	none

**Table 7:** List of highest occurring tags in decreasing order and their classification.

*n*-Gram Token	Preferred Label	Classification
device_down	device_down	Child Variable
change	maintenance	Parent/Ancestor Variable
power_outage	power_outage	Parent Variable
ups_unit	ups_unit	Parent Variable
replaced	failed	Parent/Ancestor State
crock_upgrade	crock_upgrade	Parent Variable
high_temperature_threshold_violation	high_temperature_alarm	Child Variable
high_temp_alarm	high_temperature_alarm	Child Variable

## Data Availability

Not applicable.
